# Supplementary material for: The Products of Probiotic Bacteria Effectively Treat Persistent Enterococcus faecalis Biofilms
Source: Pharmaceutics. 2022 Mar 30;14(4):751. doi: 10.3390/pharmaceutics14040751 (PMC9027392; doi:10.3390/pharmaceutics14040751)
Supplement: Supplementary file 1 [file pharmaceutics-14-00751-s001.zip › pharmaceutics-1614763-supplementary.pdf]

pharmaceutics

MDPI

Supplementary Materials

The Products of Probiotic Bacteria Effectively Treat Persistent *Enterococcus faecalis* Biofilms

Shatha Safadi, Harsh Maan, Ilana Kolodkin-Gal, Igor Tsesis and Eyal Rosen

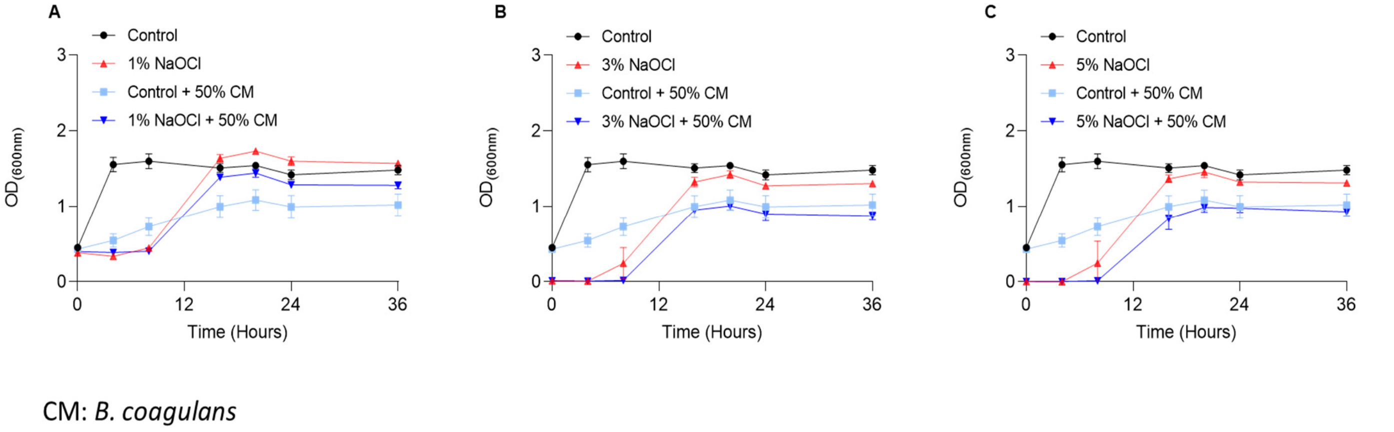

Figure S1. Secretions of the probiotic bacterium *B. coagulans* caused a delay in the regrowth of *E. faecalis* biofilms treated with sodium hypochlorite but failed to prevent it. A single colony of Strain 29212 was grown at 37 °C with shaking in liquid LB medium to a mid-logarithmic stage. Cells were diluted 1:25 into a fresh medium. Cultures were split into a 12-well polystyrene plate, 1000 μL in each well, and further incubated at 37 °C for 24 h. The established biofilms were untreated or treated with different concentrations of NaOCl (1% (A), 3%(B), 5%(C)) for 5 min. Bacterial cells were collected with 200μL PBS and diluted (1:15) in TSB-glucose medium either mixed 1:1 or not applied with *B. coagulans* condition medium. Treated cells were allowed to regrew at 37 °C with shaking. OD600 was measured manually in different time points (0h,4h,8h,16h,20h,24h,36h) using a spectrophotometer.

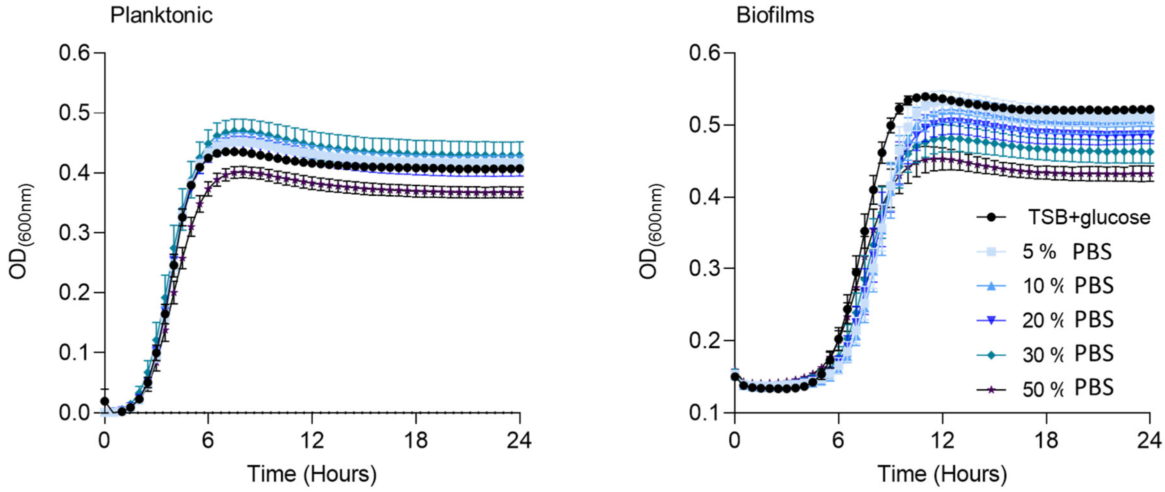

Figure S2. Planktonic and Biofilm E. faecalis growth inhibition is not due to nutrient depletion. Growth of *E. faecalis* Planktonic and Biofilm cells when exposed to PBS at indicated concentrations.
